# Non-pharmacological care for patients with generalized osteoarthritis: design of a randomized clinical trial

**DOI:** 10.1186/1471-2474-11-142

**Published:** 2010-07-01

**Authors:** Thomas J Hoogeboom, Mirelle JPM Stukstette, Rob A de Bie, Jessica Cornelissen, Alfons A den Broeder, Cornelia HM van den Ende

**Affiliations:** 1 Department of Rheumatology, Sint Maartenskliniek, Nijmegen, The Netherlands; 2 Department of Epidemiology, School for Public Health and Primary Care (Caphri), Maastricht University, Maastricht, The Netherlands

## Abstract

**Background:**

Non-pharmacological treatment (NPT) is a useful treatment option in the management of hip or knee osteoarthritis. To our knowledge however, no studies have investigated the effect of NPT in patients with generalized osteoarthritis (GOA). The primary aim of this study is to compare the effectiveness of two currently existing health care programs with different intensity and mode of delivery on daily functioning in patients with GOA. The secondary objective is to compare the cost-effectiveness of both interventions.

**Methods/Design:**

In this randomized, single blind, clinical trial with active controls, we aim to include 170 patients with GOA. The experimental intervention consist of six self-management group sessions provided by a multi-disciplinary team (occupational therapist, physiotherapist, dietician and specialized nurse). The active control group consists of two group sessions and four sessions by telephone, provided by a specialized nurse and physiotherapist. Both therapies last six weeks. Main study outcome is daily functioning during the first year after the treatment, assessed on the Health Assessment Questionnaire. Secondary outcomes are health related quality of life, specific complaints, fatigue, and costs. Illness cognitions, global perceived effect and self-efficacy, will also be assessed for a responder analysis. Outcome assessments are performed directly after the intervention, after 26 weeks and after 52 weeks.

**Discussion:**

This article describes the design of a randomized, single blind, clinical trial with a one year follow up to compare the costs and effectiveness of two non-pharmacological interventions with different modes of delivery for patients with GOA.

**Trial registration:**

Dutch Trial Register NTR2137

## Background

Osteoarthritis (OA), the most prevalent rheumatic disorder affecting the musculoskeletal system, has a major impact on functioning and independence of the elderly. In general, one distinguishes between four subgroups of peripheral OA: 1. knee, 2. hip, 3. hand and 4 generalized OA (GOA) [1]. Clinically, 10-15% of adults over 60 have symptomatic knee OA [2-5] and symptomatic hip OA occurs in 1-4% of all adults [3]. Symptomatic hand OA occurs in 10-15% of the elderly [6] and GOA in 27% of the patients with hip or knee OA [7].

Non-pharmacological treatment (NPT) is considered to be important in the management of OA in order to reduce the impact of OA on pain and physical functioning [8]. Current OA research on NPT-options, focuses mainly on the hip and knee joint [9]. An abundance of research literature illustrates that NPT is a useful treatment option in the management of hip or knee OA [9]. The initial focus of NPT should lie on self-management and patient-driven treatments, rather than on passive therapies delivered by allied health professionals [8]. Provision of information and patient education about the objectives of treatment and the importance of changes in lifestyle, exercise, pacing of activities, weight reduction and other measures to unload damaged joints is supported by two meta-analyses [10,11] on the efficacy of non-pharmacological interventions in chronic diseases. Increasing the functional capacity [12-14] and encouraging the patient to undertake and maintain regular exercise [15] have also been found effective.

There is lack of evidence concerning the optimum mode of care delivery. The more traditional face-to-face contact is by far the most evaluated type of therapy delivery. However, telephone contact aimed at promoting self-care appears to be more cost-efficient [16] and has also been associated with improvements in joint pain [17,18] and physical function [18] for up to a year in patients with knee OA. Moreover, in a recent study by Eakon et al (2009) telephone counselling was suggested a feasible mode of delivering lifestyle interventions to patients with chronic conditions and demonstrated modest improvements in diet and physical activity [19].

To our knowledge, only one study investigated the effect of a non-pharmacological intervention in the management of GOA [20]. However, in this study all GOA-patients recently underwent major joint replacement, therefore the results of this study cannot be generalized to a population in which joint replacement is not (yet) an option. Taking into account a. the extensive body of literature on NPT for hip or knee OA, b. the substantial group of patients and c. the fact OA in multiple joints is more disabling than in one joint [21-23], it is remarkable that research on the efficacy of NPT options in GOA is hitherto neglected.

Considering the latter, we infer that the development and evaluation of a treatment programme is warranted. To do so, we installed an expert group consisting of a physiotherapist, an occupational therapist, a specialized nurse, a rheumatologist and two researchers, all of whom have extensive experience with GOA patients. Consequently, the expert group systematically conceptualized a definition of GOA and a treatment programme tailored to the needs of patients with GOA and based on recommendations for the management of hip and knee OA [8,9] and on the clinical experience of the health care providers. This resulted in a best-evidence, multi-disciplinary treatment programme. Since there is no information about the optimal treatment intensity and mode of delivery, we decided to compare the effectiveness of a fully supervised multi-disciplinary program to an active control [24] (i.e. a telephone monitored program combined with two supervised contact moments). Due to the complex nature of GOA and the fact that guidelines for hip and knee OA recommend multiple NPT modalities, both interventions are multi-disciplinary [8]. We hypothesize that both programmes have beneficial effects on the patients' quality of life and ability to cope with their disease, however we expect the face-to-face programme to be superior with respect to daily functioning to the telephone programme.

The primary aim of this study is to compare the effectiveness of a supervised multi-disciplinary programme to an active control on daily functioning in patients with GOA during the first year after treatment. Secondary aims of the study are to investigate the short-term effects of interventions and to compare the cost-effectiveness of both interventions.

## Methods/Design

A pragmatic randomized, single blind, clinical, superiority trial with active controls will be used to study the aforementioned aims. The study will be performed at the outpatient rheumatology departments of the Sint Maartenskliniek Hospitals in the cities of Woerden and Nijmegen in The Netherlands. Both centres have piloted the interventions and in both centres, rooms well equipped for group based treatments are available.

**Figure 1:**
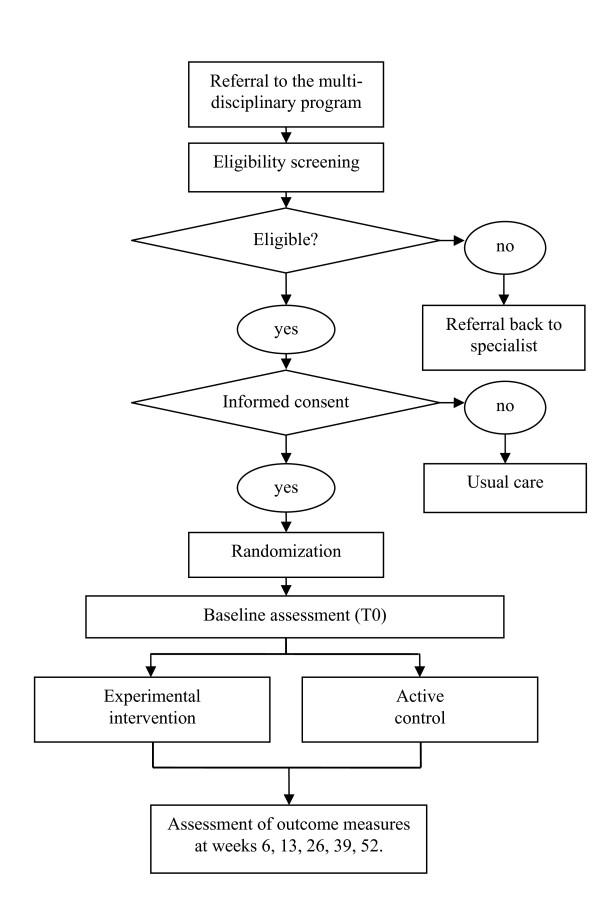
**Overview of the study design**. An overview of the study design showing recruitment, assessments, treatment groups, follow-up and outcome assessments.

Patients - referred by their rheumatologist to the outpatient department for multi-disciplinary NPT - eligible for both the GOA health care program and the trial are informed about the trial. Subsequently, consenting patients are randomly allocated to one of the two groups and followed by questionnaires for a total of 52 weeks (figure 1).

The trial has been reviewed by the Institutional Review Board of the University Medical Centre Nijmegen (protocol number 2009/290) and they concluded that the study did not fall within the remit of the Medical Research Involving Human Subjects Act. So the study can be carried out (in the Netherlands) without an approval by an accredited ethical board.

### Eligibility Criteria

Men and women (≥ 18 years old) are eligible to enter the trial if they are diagnosed with GOA (see our definition in the following paragraph), motivated to alter their lifestyle (assessed by a standardized set of questions), willing to participate in a group and able to comply with the planned time schedule of both treatment conditions. Patients are excluded when they are 1. awaiting surgery, 2. already participated unsuccessfully in a self-management program, 3. are considered not to be able to participate in a group due to limited psychological functioning (on the basis of clinical judgment of a psychologist), 4. are illiterate, 5. are not capable of communicating in Dutch or 6. are incapable of coming to the hospital.

From the patients who were in principle eligible considering the in- and exclusion criteria but decided not to participate in the study, baseline demographics (ie. age and sex) will be gathered, to assess possible selection bias.

### Definition of GOA

In the abovementioned inclusion criteria we mentioned that patients must be diagnosed with GOA. However, no uniform GOA definition is available in the literature. A pragmatic literature search elicited numerous definitions for GOA [7,25-33], mainly used in genetic studies and for the greater part based on patterns of distribution of joints with radiological changes.

In clinical practice the term GOA refers to the combination of clinical symptoms and radiographic changes in multiple joints which can be attributed to OA as obtaining a full picture of radiological changes in all joints is not feasible nor desirable in clinical practice. For the purpose of this project we formulated a pragmatic definition of GOA based upon literature findings and on the basis of consensus of several clinicians and health professionals with experience in patients with GOA. In our definition signs and symptoms are combined with radiological changes. In this project a patient is defined as having GOA if he or she complies with the following three conditions:

a. experiencing complaints in three or more groups of joints, and;

b. having at least two objective signs that indicate OA in at least two joints (objective signs indicating OA are malalignment, palpable osteophytes/nodules, crepitations over the full range of motion, and limited range of motion or radiographic signs including the presence of joint space narrowing and/or osteophytes, and;

c. is limited in daily functioning (Health Assessment Questionnaire score [34] > 0.5).

### Interventions

During a six-week treatment period, patients will receive one of the following two treatment programs:

- Interdisciplinary, group-based, self-management program (experimental intervention);

- Telephone-based, self-management program (active control).

**Figure 2:**
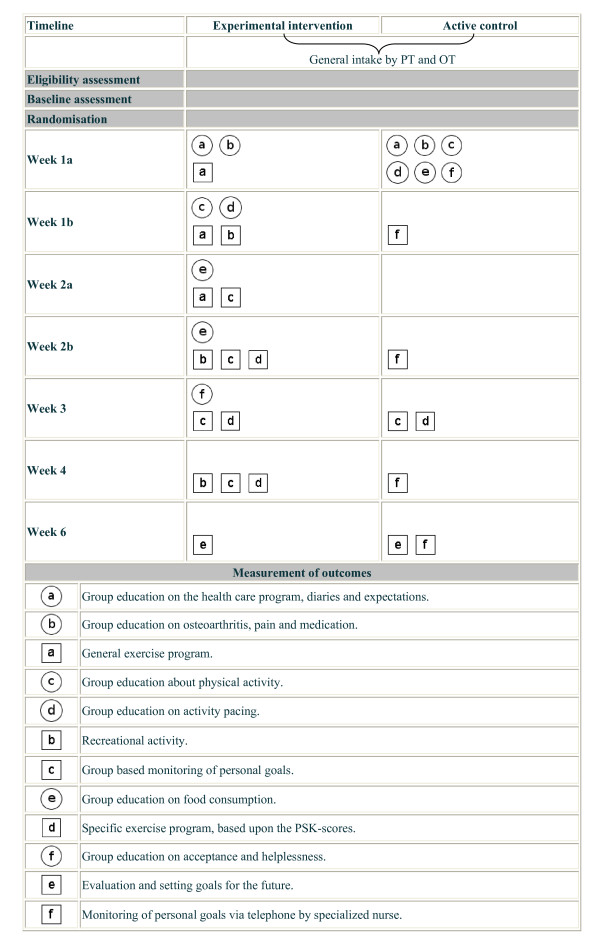
**Graphical depiction of the two self-management interventions**. Abbreviations: PSK = Patient Specific Complaints, PT = physiotherapist, OT = occupational therapist.

Both pilot tested interventions were developed from a clinical and pragmatic perspective, meaning that both interventions had to be useful and feasible in clinical practice. This resulted in two, for patients and health care providers satisfactory, interventions. However, from a research perspective lack of contrast between both interventions might be observed. To further elaborate differences between groups, we depicted a specific overview of the content of the non-pharmacological treatment in both arms in figure 2, according to the recommendations of Perera *et al *2007 [35].

For both interventions, manuals and standardized presentations were created. At baseline and every six months, all care providers assemble to assess and enhance the adherence with the treatment protocols.

#### Experimental intervention group

Patients (eight per group) allocated to the experimental intervention group attend six therapeutic group sessions and one group evaluation. During these six sessions patients aim to improve daily functioning by optimising their current lifestyle (i.e. physical activity and diet) and by enhancing self-efficacy to control the consequences of the disease in everyday life (i.e. activity pacing, pain management and daily functioning). To enhance patients' self-efficacy the 5As model of behaviour change counselling is used, which is an evidence-based approach appropriate for a broad range of different behaviours and health conditions. The 5As consists of: Assessing patient level of behaviour, beliefs and motivation; Advising the patient based upon personal health risks; Agreeing with the patient on a realistic set of goals; Assisting to anticipate barriers and develop a specific action plan; and Arranging follow-up support [36]. The following example might illustrate the use of the 5As model. A participant wears a pedometer to elicit his/her physical activity level (A1). Together with the health care provider, the patient discusses the outcome (A2) and set a goal to increase the level of physical activity (A3). Both the health care provider and the patient must believe the goal is adequate (A3) and realistic (A4). Consequently, patient and therapist closely monitor the personal goals (A5). In addition to the self-management programme, patients are also enrolled in an exercise programme aimed to 1. improve the quality of movement and 2. implement the learned exercises in the home situation.

#### Active control group

Patients enrolled in the active control group, attend two group sessions (eight patients per group) and are further monitored through four telephone contacts [37]. As per with the experimental intervention, the active control group aims to optimise the patients' current lifestyle (i.e. physical activity and diet) and to enhance the patients' self-efficacy to control the disease (i.e. activity pacing, pain management and daily functioning). Again, all patients set personal goals on the abovementioned items. Progress on these personal goals will be monitored by the health care provider through planned telephone contact. Patients are asked to self-monitor their own health-status [37], by filling out activity and dietary diaries.

### Health care providers

A total of 14 health care providers (five physiotherapists, three occupational therapists, five specialized nurses, and one dietician) are involved in the therapy sessions. All health care providers are specialized in the management of patients with musculoskeletal disorders and have experience in teaching self-management principles to groups. Moreover, all care providers took the course 'motivational interviewing'.

The experimental intervention will be provided by one of three physiotherapists, one of three occupational therapists, one of two specialized nurses and one dietician. In the active control group, the two group sessions are provided by one of two physiotherapists and two of three specialized nurses. The telephone contact will be provided by the specialized nurses. Assignment of health care providers to the therapy programs was done on basis of availability.

### Primary outcome

#### Daily functioning

The primary outcome of the study is the Stanford Health Assessment Questionnaire (HAQ) Disability Index during the first year after treatment [34,38]. The HAQ is an independent patient-reported outcome questionnaire containing 20 questions, covering eight domains of activities of daily living. For each item, there is a four-level response set that is scored from 0 to 3, with higher scores indicating more disability (0 = without any difficulty; 1 = with some difficulty; 2 = with much difficulity; and 3 = unable to do). Both total scores as well as each of the sub-scores range from 0 (no disability) to 3 (severe disability). An improvement of 0.26 points is considered to be clinically relevant between group change [39]. The HAQ has been found to be more responsive for measuring functioning than the WOMAC questionnaire; a widely used in hip and knee OA [40].

### Secondary outcomes

#### Health-related quality of life (clinical efficacy)

To assess the efficacy of the interventions on health-related quality of life (HRQoL) the RAND 36-Item Health Survey 1.0 (RAND-36) will be used [41]. Scores from the eight subscales of the RAND-36 will be aggregated into two summary scores: a Physical Component Summary (PCS) and a Mental Component Summary (MCS). This instrument has been translated and validated for use in Dutch patients [42].

#### Patients specific complaints (clinical efficacy)

Physical functioning assessed with the patient specific complaints questionnaire (PSK). The PSK is a patient-specific questionnaire in which the patient is asked to select three activities that (s)he perceives as problematic (activities that can easily be avoided are not allowed) and scores the severity on a 10 cm visual analogue scale (VAS) [43].

#### Fatigue (clinical efficacy)

Fatigue is measured with the "Subjective Fatigue" subscale of the Checklist Individual Strength (CIS) [44]. The CIS is a self-administered questionnaire assessing 20 items, concerning 4 subscales divided in: subjective experienced fatigue (8 items), concentration (5 items), motivation (4 items) and physical activity (3 items). The outcomes per question are given in a 7-point scale, ranging from the statement 'totally right' to the statement 'totally wrong'. The total score is counted in points with a range of 1-7 per question and a total score range of 8-56 points. The CIS is a sensitive instrument with good discriminating power and reliability [44].

#### Health-related quality of life (health economics)

To measure the HRQoL of patients for the purpose of economic evaluation the EuroQol-5D (EQ-5D) will be used [45]. This HRQoL instrument will be completed by the patients and is available in a validated Dutch translation. The EQ-5D is a generic HRQoL instrument comprising five domains: mobility, self-care, usual activities, pain/discomfort and anxiety/depression. The EQ-5D index is obtained by applying predetermined weights to the five domains. This index gives a societal-based global quantification of the patient's health status on a scale ranging from 0 (death) to 1 (perfect health). The utility weights captured by these preferences will enable the derivation of the Quality Adjusted Life Years (QALY) for each intervention and will be used in cost-utility analyses.

Patients will also be asked to rate their overall HRQoL on a visual analogue scale (EQ- 5D VAS) consisting of a vertical line ranging from 0 (worst imaginable health status) to 100 (best imaginable).

#### Costs (health economics)

Volumes of care will be measured prospectively using patient-based diaries (complemented by patient chart data if necessary). Per arm (intervention and control) full cost-prices will be determined using an activity based costing approach. Productivity losses for patients will be estimated using a postal questionnaire on a 3 months recall basis. The friction cost-method will be applied following the Dutch guidelines for cost analysis (Oostenbrink et al., CVZ 2004). Also travel time to a session or outpatient clinic and related costs patients make will be considered (also on the basis of 3 months recall).

The second part of the cost analysis consists of determining the cost prices for each unit of consumption in order to use these for multiplying the volumes registered for each participating patient. The Dutch guidelines for cost analyses will be used (CVZ, Oostenbrink et al., 2004). For units of care/resources where no guideline or standard prices are available real cost prices will be determined.

### Study endpoints

Participants will receive postal questionnaires at baseline, and at 6, 13, 26, 39 and 52 weeks after the start of the intervention. The primary endpoint to study the long term effects of the interventions is the averaged HAQ-score [34] as obtained from the 6, 26 and 52 week time points. The 6 week time point will provide a secondary endpoint for investigating the short term effects of the interventions. On time points 6, 13, 26, 39 and 52 costs will be assessed.

Socio-demographic information will be collected at baseline including age, gender, employment nature and body mass index. In Table 1 we outlined all outcome measures that will be collected at baseline and at follow-up evaluations.

**Table 1:** Outcome measures used at baseline and follow-up assessments

Outcome (Instrument)	Time Points (weeks)					
	**0**	**6**	**13**	**26**	**39**	**52**

						

Daily functioning (HAQ) [34]	X	X	X	X	X	X

HRQoL (RAND-36) [42]	X	X		X		X

HRQoL (EuroQol EQ-5d) [45]	X	X	X	X	X	X

Patient specific complaints (PSK) [43]	X	X		X		X

Fatigue (CIS-8) [44]	X	X		X		X

Physical activity (SQUASH) [48]	X	X		X		X

Illness Cognitions (ICQ) [65]	X	X		X		X

Patient Global Assessment (PGA) [50]		X		X		X

Self-efficacy (GSES) [51]	X	X		X		X

Kinesiophobia (TSK) [66]	X	X		X		X

Costs		X	X	X	X	X

Abbreviations: HRQoL = Health related quality of life.

### Other outcomes

Since no validated outcome measures are available yet for the assessment of health status in patients with GOA, we decided to evaluate effectiveness also with a responder analysis. We developed an adapted version of the OMERACT-OARSI Responder Criteria as the secondary outcome measure of our study [46]. This composite index permits presentation of results of symptom modifying clinical trials in OA based on individual patient responses (responder yes/no). In this study, patients are considered responders if at least 3 of the 6 targeted areas (i.e. physical functioning, pain, fatigue, physical activity, acceptance, and patient global assessment) improve by ≥ 20% [47]. We assess the targeted areas with the following secondary outcome measures: RAND-36-pain, PSK, CIS and SQUASH, ICQ, PGA (as described below).

#### Physical activity

The Short QUestionnaire to ASsess Health-enhancing physical activity (SQUASH) [48] will be used to measure physical activity. The SQUASH measures habitual physical activity level and is structured in a way that allows comparing the results to international physical activity guidelines. The questions are prestructured into activities at work, activities to/from work, household activities, leisure-time activities and sports activities. Spearman correlation has shown an overall reproducibility of 0.58 (p < 0.05) for the SQUASH. The SQUASH has been validated using an accelerometer, the CSA Inc. Activity Monitor (model AM7164-2.2), showing a Spearman correlation coefficient between CSA readings and total activity score of 0.45 (95% CI 0.17-0.66) [48].

#### Illness cognitions

Illness cognitions (acceptance and helplessness) are measured using the Illness Cognitions Questionnaire (ICQ). The ICQ is an 18-item questionnaire measuring three generic illness cognitions: helplessness, acceptance and disease benefits. Participants rate the extent to which they agree with the statements on a 4-point Likert scale, ranging from 1 (not at all) to 4 (completely). Higher scores at subscales reflect higher levels of agreement with that generic illness cognition. The scale has excellent construct and internal validity [49]. In this study we use the subscales acceptance and helplessness.

#### Global perceived improvement

Patient Global Assessment (PGA) is assessed by patients on a 8-point scale (1 = vastly worsened; 8 = completely recovered) [50].

#### Self-efficacy

Self-efficacy is evaluated with the General Self-Efficacy Scale (GSES) - the Dutch Language Version; a self-administered questionnaire assessing 10 items, concerning problems in daily living and the capability to bring up solutions for these problems [51]. The scores of the questions are rated on a 4-point scale. Possible responses are not at all true, hardly true, moderately true and exactly true yielding a total score between 10 and 40 points. A higher score represents a higher level of self-efficacy. The GSES was found to be configurally equivalent across 28 nations, and it forms only one global dimension. High reliability, stability, and construct validity of the GSES were confirmed in earlier studies [51].

### Randomization, allocation concealment and blinding

Participants included in the study are randomly assigned to one of the treatment programmes. Restricted randomization will be used by randomly varied block sizes (2 to 6) [52]. A computer-generated randomization sequence table will be produced with random allocation software [53] by an independent researcher (DJ). Subsequently an independent person will assign patients to one of the treatment groups. This person has no information about the persons included and has no influence on the assignment sequence or on the decision about eligibility of patients.

Patients and health care providers allocated to the experimental and active control group will be aware of the allocated arm, whereas the outcome assessor and data analysts will be kept blinded to the allocation.

### Sample size

For the sample size calculation we used the statistical package G*power 3.0.10 [54]. We utilized the equation for sample size required per group using an unpaired t-test to compare differences between two independent means. To detect a minimal clinically important change of 0.26 points [39] in mean HAQ scores between both groups, assuming a SD of: 0.66 (SE*√N = 0.04*√271 = 0.66) [40] with 80% power and a two-sided 5% level, we will need 102 patients per arm (effect size is 0.26/0.66 = 0.39). The abovementioned sample size calculation is relevant for analyses with independent t-tests.

In our analyses, however, we will use the baseline HAQ as a covariate. By a straightforward generalisation of the method described in Borm et al [55], it can be shown that in this case the sample size must be multiplied by (1-(k-1)ρ)/k - ρ_B_^2 ^(see note below), where k is the number of follow-up assessments (3 in our case), where ρ_B _is the correlation between the outcome measured at baseline and at follow-up and where *p *is the correlation between the follow-up measurements. Although some publications report test-rest correlation for the HAQ of more than 0.8 (31), there is no direct information about the correlation that is to be expected in our trial. We expect ρ_B _to be smaller than *p *(even within the treatment groups), because the interventions will be 'between' the baseline and follow-up assessments. The interventions may not have the same effect on all patients and therefore decrease the correlation. When *p *is between 0.7 and 0.9 and ρ_B _= p-0.2, the sample size can be reduced by a (design) factor 0.44 to 0.55. For p between 0.8 and 0.9 and ρ_B _= *p*-0.1, the sample size can be reduced by a factor 0.38 to 0.44. A trial with 55 patients per treatment group will then have at least 80% power (when the design factor is 0.55). In the most optimistic scenario, when the design factor is 0.38, the study has slightly over 90% power.

Finally, as the patients will be treated in groups (cluster) of approximately eight, the patient numbers have to be increased by a factor 1+(8 -1)*ICC. For ICC = 0.05, this leads to 74 patients. In order to compensate for possible drop-outs (15%), we plan to enrol 85 patients per treatment group.

Note:

When Y = mean(Y_1_, ...Y_k_) and the baseline and follow-up measurements Y_i _all have standard deviation σ, the variance of Y is ((1+(k-1)*p*)/k)σ^2^, the correlation between B and Y is k ρ_B_/sqrt(k+(k(k-1) p) and the formula in Borm *et al *yields the design factor (1-(k-1)*ρ*)/k - ρ_B_^2^.

### Planned data analysis

Study data are entered in Access 2003, exported to the statistical package STATA v10 stored on a secure network drive. Five percent of the data will be entered twice to assess percentage and nature of typing errors. All paper records are stored in a locked cabinet in an anonymised format. The researcher will check for any missing data and will manage this according to the recommendations of the questionnaires. Descriptive statistics will be used to determine participant characteristics. Continuous variables will be reported using means, standard deviations (SD) and inter-quartile ranges when appropriate, if not median and ranges are shown. For dichotomous/categorical variables, we will display absolute numbers and percentages. The primary analysis will be according to the intention to treat principle.

#### Clinical efficacy

The primary variable, HAQ during the first year after treatment, will be analysed with a random effects model with the HAQ scores after 6, 26 and 52 weeks as dependent variable. The fixed factors will be assessment (6, 26 or 52 weeks), treatment group, sex and baseline value. In order to account for the group wise treatment and the repeated measurements, the random effects group and patient will be included.

HAQ immediately after treatment will be evaluated in a random effects model with fixed factors treatment group, sex and baseline value, and random factor group. All other continuous variables will be analysed in a similar way. Skewed variables will be transformed before analysis. For dichotomous outcomes, random effects general linear models with Bernoulli distribution and linear link function will be used, similar to the ones described for continuous outcomes.

Changes in effect size over time will be evaluated by adding the interaction of assessment and treatment group to the model.

#### Health economics

The economic evaluation is based on the general principles of a cost-effectiveness analysis and cost-utility analysis. For the cost-effectiveness analysis we will calculate the incremental cost-effectiveness ratio (ICER) as cost per unit improvement on the HAQ. For the cost-utility analysis we will calculate the ICER as cost per Qaly gained. This ICER will be evaluated stochastically and uncertainty will be determined using the bootstrap method and/or Fieller method. A cost-effectiveness acceptability curve will be derived that is able to evaluate efficiency by using different thresholds (Willingness To Pay) for a QALY. The impact of uncertainty surrounding deterministic parameters (for example cost-prices) on the ICER will be explored using one-way sensitivity analyses on the range of extremes. The economic evaluation is done along-side the clinical trial and consequently adheres to the earlier presented design.

## Discussion

To date, research on NPT options for OA has mainly focused upon patients with hip and knee OA. In 2008, NICE disseminated multiple recommendations for future OA research based on the research hiatuses they identified. One of their research questions was: "What are the benefits of individual and combination OA therapies in people with multiple joint region pain?". This study will contribute to the body of evidence on NPT in GOA patients.

A possible limitation in our study is the limited contrast in the content of the experimental and control intervention. Both interventions were developed from a clinical and pragmatic perspective. Since both interventions should be directly implementable in clinical practice after study completion, we created two treatment protocols according to the recommendations outlined in OA guidelines and current best-practice. The content of both interventions is very similar but several critical differences are apparent such as the mode of delivery, the number of involved health care providers and the number of group-sessions. Specific insights in the effectiveness and costs of these differences will aid health care providers and care vendors in their decision making for the management of patients with GOA.

To our knowledge we are the first to define GOA from a clinical rather than a radiographic perspective, as no consistent clinical useful definition of GOA is available. In 1952, Kellgren and Moore defined GOA as involvement of multiple joints combined with Heberden's nodule [31]. Since then, multiple definitions of GAO have been used, for the greater part based on radiological changes. Most definitions state that GOA involves at least three joints [31], although this again is questioned [27]. The group of joints most often incorporated in definitions are the hands, neck, lower back, knees and hips [7,56,57], whereas other definitions postulate that the involvement of atypical joints [25,26] or hallux valgus [25,58] is essential for GOA. To date, two specific phenotypes of GOA have been established [28], however, these phenotypes are far from useful in daily practice as these phenotypes only represent a very small proportion of patients with OA-like complaints in multiple joints. Considering the low feasibility and desirability of obtaining radiographs of a large number of joint in clinical practice, we believe that clinical signs and symptoms should also be taken into account in the definition of GOA. Especially, since pain at multiple joint sites is associated with lower levels of functioning [21,23,59-61], more pain [21,23,59,60] and higher levels of distress [21,59,62], and complaints rather than radiographic OA are the main motivation for patients to engage in therapy. So, for the purpose of this project we formulated a pragmatic definition of GOA (as described earlier) based upon literature findings and on the basis of consensus of several clinicians and health professionals with experience with patients with GOA.

There is a need for outcome measures to evaluate self-management interventions [63]. Self-management is defined as the individual's ability to manage the symptoms, treatment, physical and psychosocial consequences and lifestyle changes inherent in living with a chronic condition [64]. Characteristically, one or more of these areas are addressed by self-management interventions [63]. In our study we target physical functioning, pain, fatigue, physical activity, and acceptance. However, no comprehensive outcome measure is available to measure all these different aspects. Mulligan *et al *(2005) state that when designing a self-management intervention, it is important to be clear about what the intervention is designed to achieve, in what areas it is likely to have an effect, and to choose outcome measures accordingly [63]. Therefore, we decided to include a responder analysis - derived from the OMERACT-OARSI responder criteria [46] - as one of the secondary measures in our analysis that specifically evaluates those areas we aim to address. In a future publication we intend to evaluate and discuss this method of assessing self-management interventions.

In conclusion, this study will provide additional insights in the effectiveness of non-pharmacological interventions for GOA. The publication of our study protocol enables future readers to compare what was originally intended with what was actually done, thus preventing both "data dredging" and post-hoc revisions of study aims.

## Competing interests

The authors declare that they have no competing interests.

## Authors' contributions

Authors TJH, MJPMS, RAB, JC, AAB, CHME; 1) have all contributed to conception and design of this trial; 2) have been involved in drafting the manuscript and revising it critically for important intellectual content; and 3) have given final approval of this version to be published.

## Pre-publication history

The pre-publication history for this paper can be accessed here:

http://www.biomedcentral.com/1471-2474/11/142/prepub
